# Supplementary material for: Laser-Induced Graphene Heater Pad for De-Icing
Source: Nanomaterials (Basel). 2021 Nov 16;11(11):3093. doi: 10.3390/nano11113093 (PMC8619929; doi:10.3390/nano11113093)
Supplement: Supplementary file 1 [file nanomaterials-11-03093-s001.zip › nanomaterials-1443288-supplementary.pdf]

nanomaterials

Supplementary Material

Laser-Induced Graphene Heater Pad for De-Icing

Jun-Uk Lee [1,†], Chan-Woo Lee [1,†], Su-Chan Cho [1,†] and Bo-Sung Shin [2,*]

[1] Department of Cogno-Mechatronics Engineering, Pusan National University, Pusan 46241, Korea; lju3534@naver.com (J.-U.L.); cwleeho2@naver.com (C.-W.L.); cho_brian@naver.com (S.-C.C.)
[2] Department of Optics and Mechatronics Engineering, Pusan National University, Pusan 46241, Korea
* Correspondence: shinbs7123@gmail.com; Tel.: +82-51-510-2787
† These authors contributed equally to this work.

Table S1 shows our specifications of the 355 nm UV pulsed laser.

Table S1. Specifications of the 355 nm UV pulsed laser.

Parameter	Unit	Value
Wavelength	nm	355
Average power	Watt	~1.5
Pulse duration	ns	25
Repetition rate	kHz	30
Mode	-	TEM_{00}
Beam diameter	mm	1.5
Beam divergence	mard	<0.5

Table S2 summarizes our laser beam condition (laser speed, spot size, dynamic fluence, overlapping factor).

Table S2. Laser beam condition.

Laser Speed (mm s^{-1})	Spot Size (μm)	Dynamic Fluence (J/cm^2)	Overlap Factor (O_f)
100	70	18.57	99.995
80	75	21.66	99.996
60	80	27.08	99.997
40	95	34.21	99.998
20	120	54.16	99.999

For a given laser power, P [W], and scanning speed, U [mm/s], a dynamic fluence, F [J/mm^2], can be defined as [1]:

$$F = \frac{P}{DU} \tag{1}$$

where D [mm] is the laser beam spot diameter. The product DU in equation corresponds to the area exposed by the effective focal spot as the laser head moves along a predefined path.

Overlapping factor can be calculated as [2]:

$$O_f = \left(1 - \frac{v/f}{D + vt}\right) \times 100 \tag{2}$$

where O_f is the overlapping factor, D is the laser beam diameter on the work piece, t is the pulse duration (ms), f is the pulse frequency (Hz), and v refers to the laser speed (mm/s).

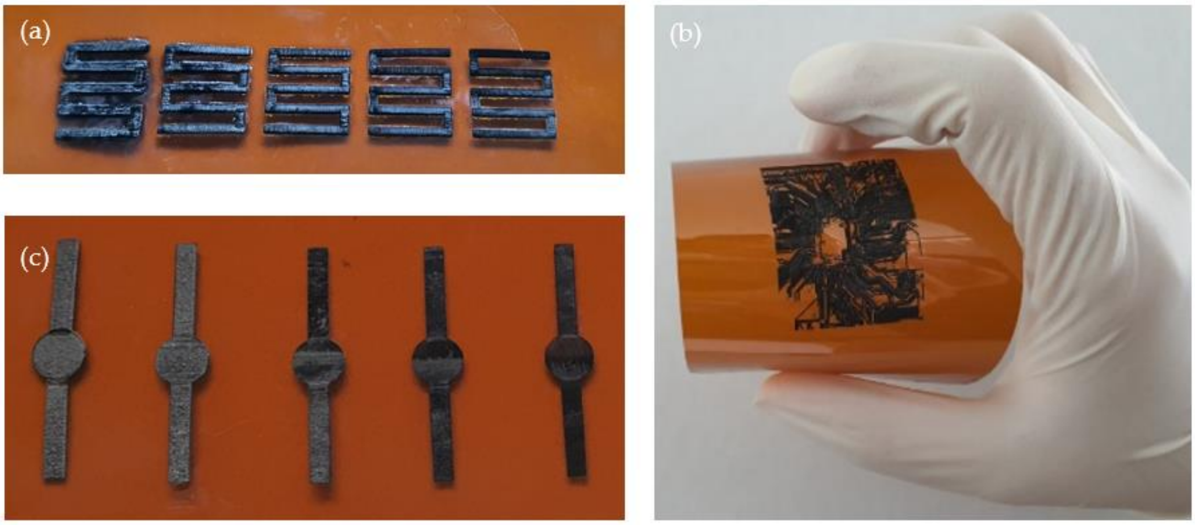

Figure S1. The various LIG patterns fabricated on polyimide; (**a**) Heater patterns on PI at scanning speed of 20, 40, 60, 80, and 100 mm/s, (**b**) Printed circuit board patterns on PI at the bending state, (**c**) Electrode patterns fabricated at scanning speed of 20, 40, 60, 80, and 100 mm/s. The LIG patterns can be utilized as various electronic devices. They show good performance at the bending state.

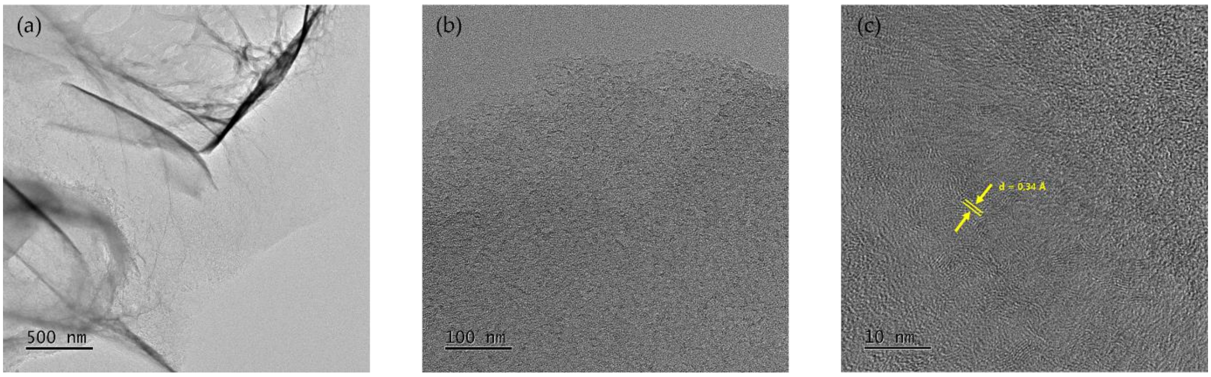

Figure S2. TEM images of LIG patterns fabricated at scanning speed of 60 mm/s; (**a**) 500 nm, (**b**) 100 nm, (**c**) 10 nm scale bar. TEM was employed to characterize the nanostructure on LIG patterns. The average d-spacing of 0.34 Å is indicated in Figure S4a–d [3].

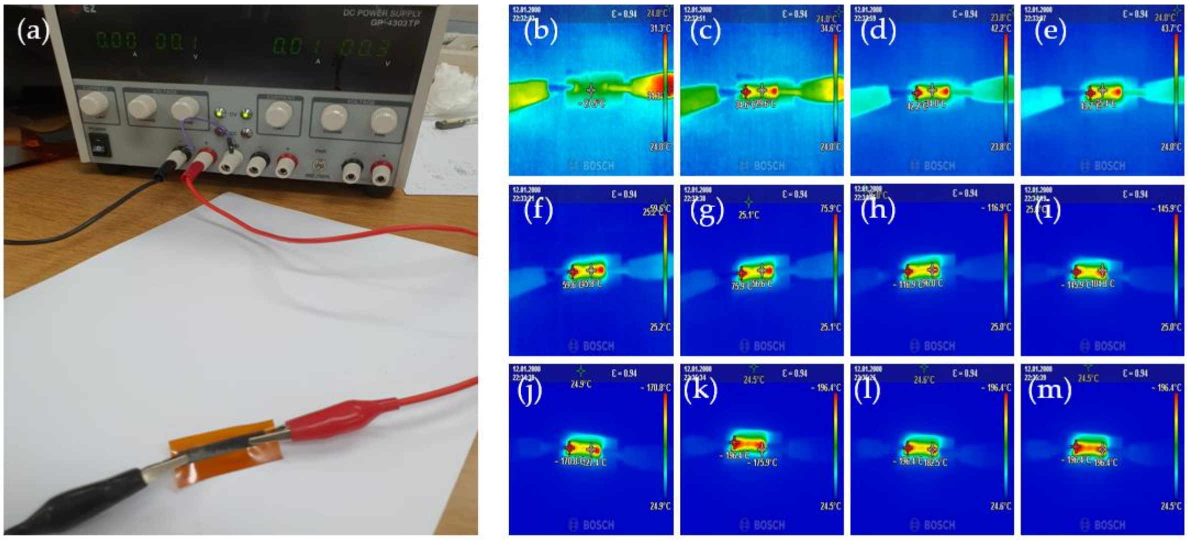

Figure S3. Electrothermal test of the flexible LIG heater (150 mm^2) without silver paste; (a) 1–12V voltage applied to LIG heater using the power supply, (**b**–**m**) Infrared image of the LIG heater (27.5–196.4 °C). The voltage was gradually increased and applied to the LIG heater sample where the silver paste was not attached (Figure S3 **a**). As shown in Figure S3**b**–**m**, contact resistance occurs in the LIG heater. Since the contact resistance between our LIG pattern and the probe is large, temperature distribution is not evenly distributed according to the Joule's law [4]. (Figure S3**b**–**m**). In order to make a heater have a uniform heat distribution characteristic, the contact resistance must be reduced. We expect that it is a phenomenon in which heat is not easily generated in the center because conduction based on Simmons equation occurs by the 3D graphene inside.

The Simmons equation is defined as [5]:

$$R_{tunnel} = \frac{V}{AJ} = \frac{h^2 d}{Ae^2\sqrt{2m\lambda}} \cdot \exp\left(\frac{4\pi d}{h}\sqrt{2m\lambda}\right) \quad (3)$$

where J is the tunneling current density; V is the electrical potential difference; A is the cross-sectional area of the tunnel; e is the quantum value of electricity; m is the mass of an electron; h is Planck's constant; d is the distance between conductive particles; and λ is the height of the energy barrier.

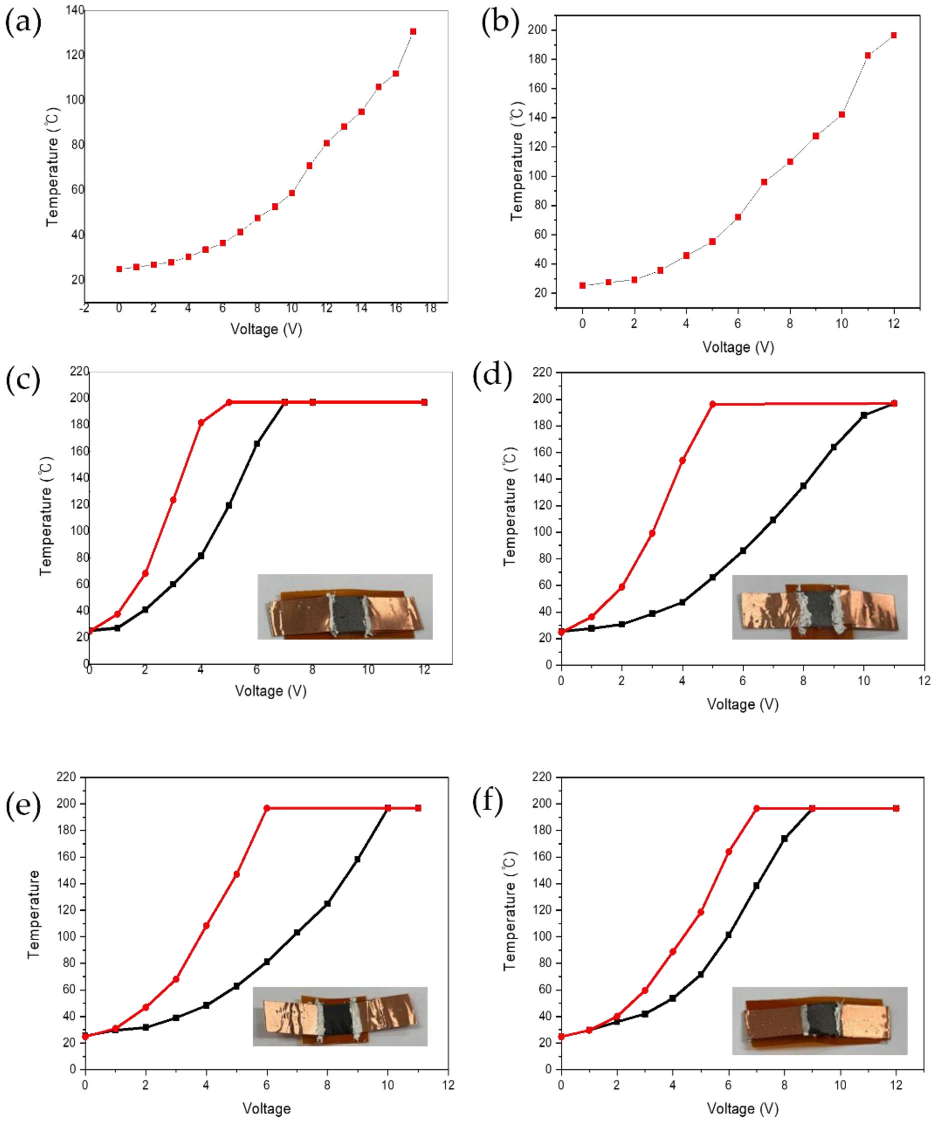

Figure S4. The temperature–voltage curve of the LIG heater; (**a**) Sample size 40mm x 2.5mm LIG heater, and (**b**) Sample size 30mm x 5mm LIG heater. The temperature–voltage curve for the same sample with the existence of silver paste at (**c**) scanning speed of 20 mm/s, (**d**) scanning speed of 40 mm/s, (**e**) scanning speed of 60 mm/s, and (**f**) scanning speed of 80 mm/s. When the voltage was applied, the temperature on the surface of the LIG heater started to increase until reaching satura-tion temperature. As shown in Figure S5**a**–**f**, the higher the voltage applied to the LIG heater was, the higher the saturation temperature was. For the samples with silver paste, the saturation temper-ature is reached with less voltage applied compared to patterns without silver paste (Figure S5**c**–**f**).

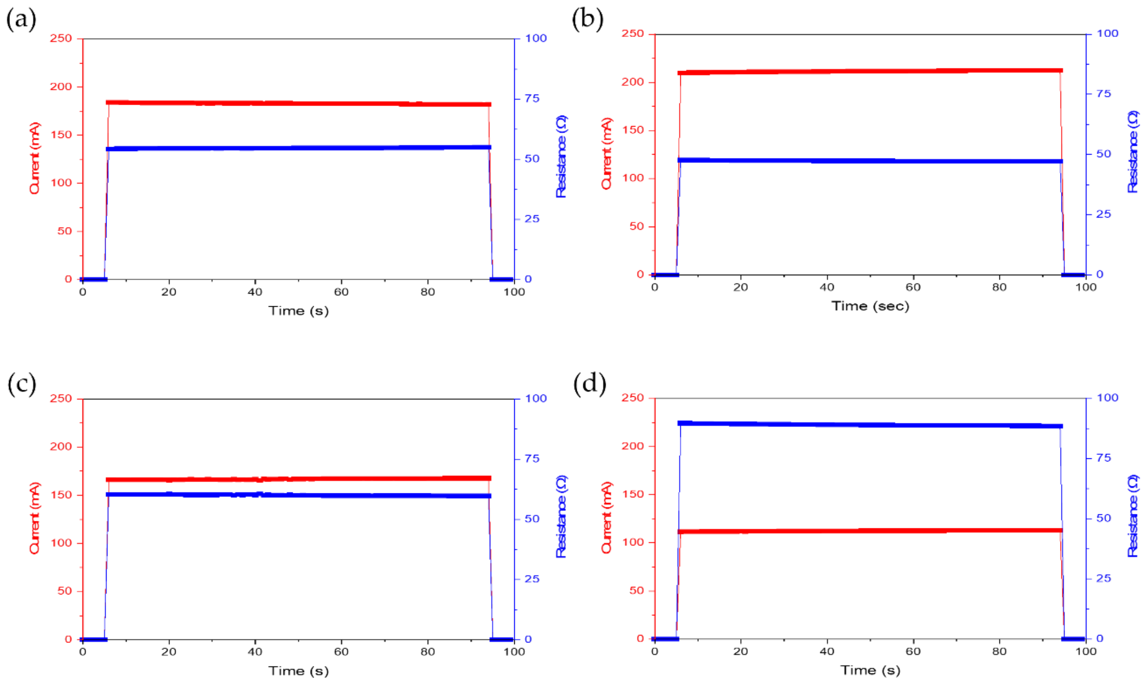

Figure S5. Resistance (blue) and current (red) over time speed for voltages of 10 V: LIG heater fabricated according to laser scanning (**a**) 20 mm/s, (**b**) 40 mm/s, (**c**) 60 mm/s, and (**d**) 80 mm/s. As shown in Figure S6, the heater resistance and current could be considered constant when fixed voltages of 10 V were applied between its terminals for almost 70 s [6]. It could be seen that our LIG patterns show good ohm's law.

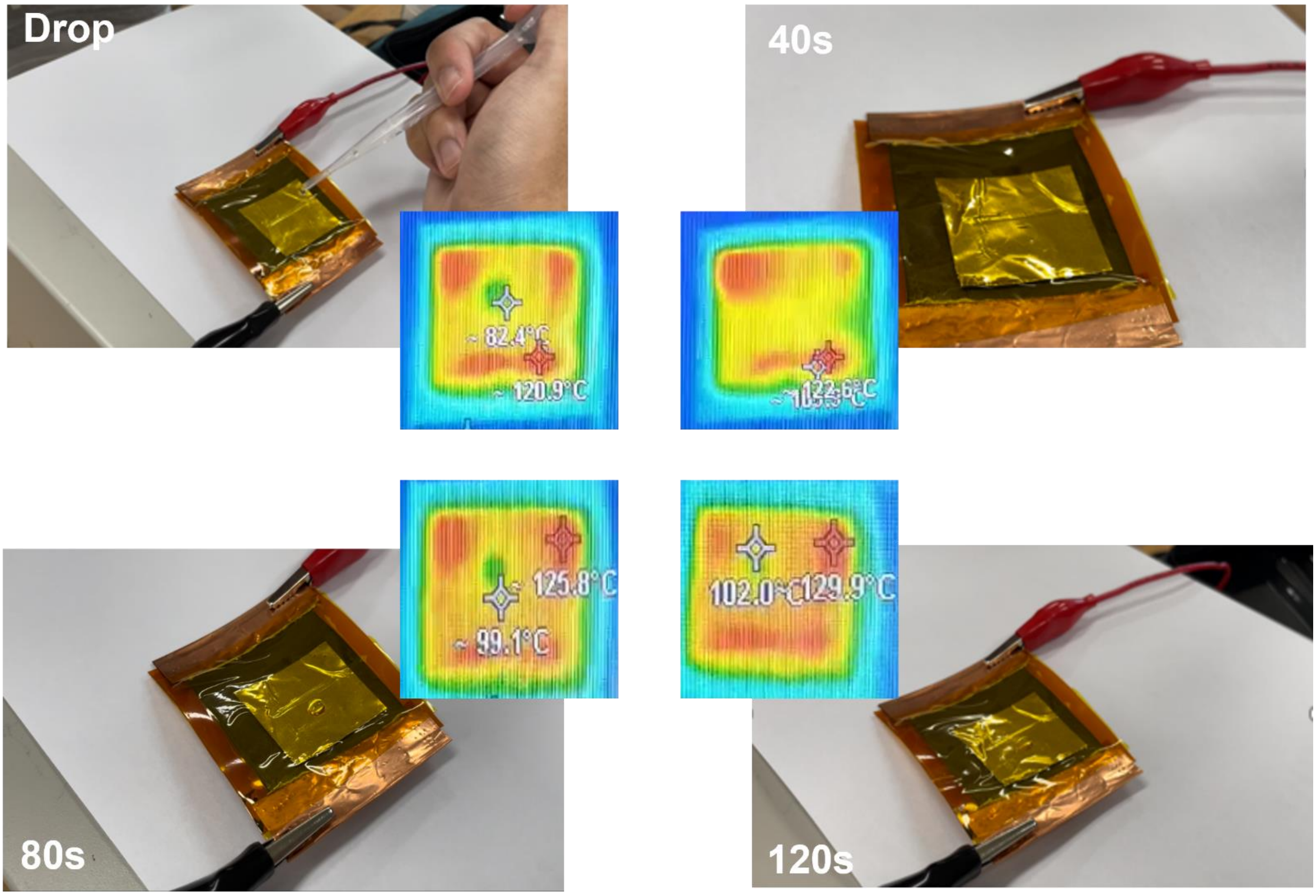

Figure S6. Evaporation test of water droplet (0.05 mL) with LIG heater pad/aluminum foil. To confirm the thermal conductivity between LIG pattern and metal film, we demonstrated a further experiment with a drop of water (0.05 mL) located on the LIG heater pad/aluminum foil in Figure S7.

The effective thermal (and electrical) conductivities of the graphitic structure can be estimated by using the Rayleigh equation, which is applicable for an isotropic medium with large void fractions [1].

$$\frac{K_{eff}}{K_0} = 1 + \frac{3\theta}{\left(\frac{k_1 + 2k_0}{k_1 - k_0}\right) - \theta + 1.569\left(\frac{k_1 - k_0}{3k_1 - 4k_0}\right)\theta^{\frac{10}{3}}} \quad (4)$$

where K_{eff} is the effective thermal conductivity of the porous medium and K_0 and k_1 are the thermal conductivities of carbonized PI and voids filled air, respectively. θ is the volume fraction of the air gaps in the structure.

Aluminum foil was selected for an optimal substrate with outstanding thermal conductivity. We could observe the temperature and state change of the water droplet. It was confirmed that the size of the water droplet was gradually decreased with 12 V applied. After 120 s, the water droplets on the heater were completely evaporated. These results show that our LIG patterns have good thermal conduction to aluminum foil [7].

Reference

1. Karimi, G.; Lau, I.; Fowler, M.; Pope, M. Parametric study of laser-induced graphene conductive traces and their application as flexible heaters. *Int. J. Energy Res.* **2021**, *45*, 13712–13725, https://doi.org/10.1002/er.6701.
2. Lee, J.-U.; Ma, Y.-W.; Jeong, S.-Y.; Shin, B.-S. Direct Fabrication of Ultra-Sensitive Humidity Sensor Based on Hair-Like Laser-Induced Graphene Patterns. *Micromachines* **2020**, *11*, 476, https://doi.org/10.3390/mi11050476.
3. Wang, C.; Song, M.; Chen, X.; Li, D.; Xia, W.; Xia, W. Effects of Buffer Gases on Graphene Flakes Synthesis in Thermal Plasma Process at Atmospheric Pressure. *Nanomaterials* **2020**, *10*, 309, https://doi.org/10.3390/nano10020309.

4. Noh, S.H.; Eom, W.; Lee, W.J.; Park, H.; Ambade, S.; Kim, S.O.; Han, T.H. Joule heating-induced sp2-restoration in graphene fibers. *Carbon* **2018**, *142*, 230–237, https://doi.org/10.1016/j.carbon.2018.10.041.
5. Jeong, S.-Y.; Lee, J.-U.; Hong, S.-M.; Lee, C.-W.; Hwang, S.-H.; Cho, S.-C.; Shin, B.-S. Highly Skin-Conformal Laser-Induced Graphene-Based Human Motion Monitoring Sensor. *Nanomaterials* **2021**, *11*, 951, https://doi.org/10.3390/nano11040951.
6. Romero, F.J.; Rivadeneyra, A.; Ortiz-Gomez, I.; Salinas, A.; Godoy, A.; Morales, D.P.; Rodriguez, N. Inexpensive Graphene Oxide Heaters Lithographed by Laser.. *Nanomaterials* **2019**, *9*, 1184, https://doi.org/10.3390/nano9091184.
7. Zhang, L.; Hou, G.; Zhai, W.; Ai, Q.; Feng, J.; Zhang, L.; Si, P.; Ci, L. Aluminum/graphene composites with enhanced heat-dissipation properties by in-situ reduction of graphene oxide on aluminum particles. *J. Alloy. Compd.* **2018**, *748*, 854–860, https://doi.org/10.1016/j.jallcom.2018.03.237.
